# Benefits and Reduced Hospital Costs of Direct Surgery in Perforated Appendicitis With Abscess Cost-effectiveness Analysis of Treatment Complicated Appendicitis

**DOI:** 10.1097/SLE.0000000000000968

**Published:** 2021-07-07

**Authors:** Euitae Kim, Kilhwan Kim, Yoonjoon Park

**Affiliations:** Department of Surgery, Dankook University College of Medicine, Cheonan, Republic of Korea

**Keywords:** perforated appendicitis, interval appendectomy, direct surgery, cost-benefit analysis

## Abstract

Elective interval appendectomy (IA) after percutaneous catheter drainage (PCD) is traditionally advocated for perforated appendicitis with an abscess. However, this is not the only way to manage these patients when we consider the cost-effectiveness of treatment. This study compared the outcomes and cost-effectiveness of patients who underwent IA and those who underwent direct surgery.

**Materials and Methods:**

A retrospective analysis of 79 patients who were diagnosed with perforated appendicitis with abscess was conducted. We compared the hospital course, outcomes, and total medical costs between the 2 groups.

**Results:**

Forty-three patients underwent PCD insertion for the management of appendiceal abscess (IA group), and 36 underwent appendectomy (DS group). There was no significant difference in abscess size (5.67 vs. 5.35 cm, *P*=0.15), appendectomy method (laparoscopic/open 39/4 vs. 37/5, *P*=0.523), or complications (7 vs. 6 cases, *P*=0.963) between the 2 groups. The operation time was longer in the DS group (83.8 vs. 112.7 min, *P*<0.001). However, length of hospitalization (15.4 vs. 7.7 d, *P*<0.001) and total hospital cost (US$2090.47 vs. US$3402.22, *P*<0.001) was greater in the IA group.

**Conclusion:**

Direct surgery without PCD insertion in perforated appendicitis accompanied by abscess is more cost-effective and reduces the total length of hospitalization compared with the traditional IA.

Appendicitis is one of the most common causes of abdominal surgical emergencies worldwide. Globally, about 1 in 10 people visit the hospital because of acute appendicitis.^1^ The causes of appendicitis were thought to be inflammation of the appendiceal lumen due to the obstruction which is originated by a fecalith, lymphoid hyperplasia, rare parasitic infection, or a tumor. The overall incidences of appendicitis and perforated appendicitis were 22.71 and 2.91 per 10,000 population per year, respectively.^2^,^3^ Appendicitis is divided into uncomplicated and complicated appendicitis. Uncomplicated appendicitis is an early stage that is not accompanied by any signs of perforation, abscess, or phlegmon. Complicated appendicitis is accompanied by abscess formation or phlegmon, which is the result of perforation of the inflamed appendix.^4^ Usually, the mortality rate associated with appendectomy with uncomplicated appendicitis is very low, and the complication rate after surgery is 5% to 28%.^5^ But the immediate appendectomy for patients with complicated appendicitis remains controversial because of the intra-abdominal abscess, wound-site infection, and difficulties in closing the stump of the appendix after laparoscopic appendectomy.^6^ Typically, surgeons consider nonoperative treatment by inserting a percutaneous drainage and antibiotics followed by interval appendectomy (IA).^7^ However, this approach would require multiple hospital readmissions and outpatient visits compared with those who had immediate appendectomy alone. The aim of this study is to determine whether direct surgery can be considered as a safer and more cost-effective option to traditional IA with percutaneous catheter drainage (PCD) in the management of perforated appendicitis with an abscess.

## MATERIALS AND METHODS

We retrospectively reviewed all patients with a diagnosis of acute appendicitis at a single university-affiliated community hospital between January 2012 and December 2018. Among patients diagnosed with acute appendicitis, we enrolled the patients who underwent surgery and with an abscess cavity (organized fluid collection or complicated fluid collection) confirmed by an abdominopelvic computed tomography (A-P CT) scan or abdominal ultrasound only. We excluded the patients who were confirmed perforated appendicitis after the operation.

All consecutive patients who had an appendiceal abscess and underwent surgery. Patients were divided in 2 groups. One group underwent PCD insertion for abscess, received intravenous or oral antibiotics and underwent an IA after the abscess and inflammation subsided (IA group). The other group underwent direct surgery after perforated appendicitis and the abscess cavity was confirmed (DS group). Patients who did not undergo IA after abscess drainage with PCD insertion and antibiotics were excluded.

The IA group underwent PCD insertion by an on-duty radiologist after confirmation of the abscess. Then, they were admitted for antibiotic management and monitoring of their PCD. After resolution of patients’ symptoms and decrease in PCD drainage, the patients were discharged and their PCD was removed. A routine checkup [1 to 2 wk after discharge and 4 to 10 wk after first outpatient department (OPD) visit] and follow-up radiologic examination(before determining operation) were conducted. If the patients did not have any symptoms of abscess recurrence or signs of peritoneal irritation during the observation period after hospital discharge, they were admitted for planned surgery about 8 to 12 weeks later.

The DS group underwent surgery because PCD insertion was not available. Because the small bowel is anterior to the abscess blocking the access to the abscess cavity or the blood vessel was positioned around the abscess cavity. Or, if the surgeon thought that the patients were not enough to stand the period of medical care considering the patients’ comorbidities and general condition. For example, a patient who is taking chemotherapy could not stop the treatment during the control of the inflammation with antibiotics. Or some patients could not stop the medication because of comorbidities. We retrospectively analyzed the medical records of the patients. We compared the clinical course, outcomes, and total medical costs of patients who underwent IA after antibiotic management and PCD insertion for the abscess cavity and those of patients who underwent direct surgery after diagnosis. The size of the abscess cavity was based on the reading records of radiologists. The total cost of treatment during hospitalization was calculated as all expenses incurred in treating the patients from the date of admission to the date of discharge. The total cost of the OPD visits was calculated by summing up the cost incurred per day. The costs of additional OPD visits or hospitalization due to relapse of symptoms and complications during the observation period were also included in the respective care costs. We analyzed the hospitalization period, surgical method, surgical time, PCD intervention period, antibiotic use period, postoperative drain insertion, complications of treatment (wound abscess, intra-abdominal abscess recurrence, rehospitalization, and mortality) of the DS and IA groups. We also compared the total length of hospitalization, number of OPD visits, costs of the hospitalization, and const of OPD visits of the 2 groups.

Statistical analysis was performed using SPSS version 19 (IBM Corp., Armonk, NY). The study data were evaluated using descriptive statistical methods such as mean and SD, median, frequency, ratio, and minimum-maximum. The quantitative data of the 2 groups were compared using the Student *t* test for data with normal distribution, and using the Mann-Whitney *U* test for data with non-normal distribution. Qualitative data were compared using the Pearson χ^2^ and Fisher exact tests. *P*-values of <0.05 were considered statistically significant.

## RESULTS

The 79 patients were enrolled in this study and we divided the patients in 2 groups. The IA group patients were 43 and the DS group patients were 36. Table 1 shows the baseline characteristics of the IA and DS groups. The IA and DS groups consisted of 43 and 36 patients, respectively. There was no statistically significant difference between the groups in terms of age and sex distribution (both *P*>0.05). In addition, there were no significant differences in the American Society of Anesthesthesiologist Physical Status Classification System (ASA) and Eastern Cooperative Oncology Group (ECOG) scores between the 2 groups (*P*>0.05, booth). The initial abscess size, which was measured during the radiologic exam, showed no statistically significant difference (*P*=0.555).

**TABLE 1:** Demographics and Patient Characteristics

	Group IA (Interval Appendectomy)	Group DS (Direct Surgery)	*P*
Number, n (%)	43	36	
Age (y)	5-85 (50)	2-90 (45)	0.325
	48.65±20.27	43.67±24.49	
Sex, n (%)			
Male	26 (60.47)	19 (52.78)	0.505
Female	17 (39.53)	17 (47.22)	
ASA score, n (%)			0.341
I	27 (62.80)	19 (52.80)	
II	13 (30.20)	13 (36.10)	
III-VI	3 (7.00)	4 (11.10)	
ECOG score, n (%)			0.25
0	36 (83.70)	33 (91.67)	
1	4 (9.30)	2 (5.56)	
2	2 (4.70)	1 (2.78)	
3-5	1 (2.30)	0	

The hospital stay after surgery was longer in the DS group than in the IA group (*P*<0.001). However, after including the duration of hospitalization after PCD insertion and for antibiotic management, the total length of hospitalization was significantly longer in the IA group (*P*<0.001). Moreover, the IA group visited the outpatient department more than the DS group (*P*<0.001). However, OPD visit counts were almost the same after surgery (*P*=0.960). The 2 groups showed no significant differences in choosing the operation method between laparoscopic and open surgery (*P*=0.523). Operating time and blood loss were higher for the DS group (*P*<0.001). The duration of antibiotic therapy was significantly longer in the IA group (*P*<0.001) (Table 2).

**TABLE 2:** Analysis of Outcome

	Group IA (Interval Appendectomy)	Group DS (Direct Surgery)	*P*
Days of admission (d)			
PCD insertion and antibiotics	11.07±6.92		
For Surgery	4.84±1.72	7.69±2.71	<0.001
Total admission days	15.40±7.14	7.69±2.71	<0.001
OPD visit			
After PCD insertion and antibiotics	2.16±1.75		
After surgery	1.33±0.52	1.33±0.83	0.96
Total days of OPD visit	3.49±1.72	1.33±0.83	<0.001
Method of surgery, n (%)			
Laparoscopic	39(90.70)	31(86.11)	0.523
Open	4(9.30)	5(13.89)	
Operating time (min)	83.84±31.32	112.72±37.48	<0.001
Blood loss (mL)	6.05±5.59	20.97±12.92	<0.001
Duration of using antibiotics*			
PCD insertion and medical care	22.42±12.81		
After surgery	4.05±2.73	12.92±6.45	0.55
Total days of antibiotics using	26.47±13.37	12.92±6.45	<0.001

* Intravenous and oral antibiotics.

OPD indicates outpatient department; PCD, percutaneous catheter drainage.

The cost of hospitalization after surgery was higher in the DS group than in the IA group (*P*<0.001). However, the total cost of hospitalization, which included the cost of the IA group’s admission period for PCD insertion or antibiotic management, was higher in the IA group than in the DS group (*P*<0.001). In addition, the IA group spent more on OPD visits (*P*<0.001) (Table 3). The ratio of the observed morbidity did not differ significantly between the 2 groups (*P*=0.963) (Table 4). No surgery-related mortalities were observed during the observation period.

**TABLE 3:** Analysis about Cost-effectiveness of Admission and OPD

	Group IA (Interval Appendectomy)	Group DS (Direct Surgery)	*P*
Cost of admission (US$)*			
Admisson for PCD insertion and antibiotics	2919.08±1955.56		
Admission for surgery	2179.63±633.72	3402.22±1042.95	<0.001
Total cost of admission	5090.47±2066.31	3402.22±1042.95	<0.001
Cost of OPD visit (US$)*	323.32±249.79	61.25±64.09	<0.001

* 1US$=1200KRW.

OPD indicates outpatient department; PCD, percutaneous catheter drainage.

**TABLE 4:** Complications

	Group IA (Interval Appendectomy) (n=43)	Group DS (Direct Surgery) (n=36)	*P*
Frequency of diseases	7 (16.28%)	6 (16.67%)	0.963
Readmission after discharge	2	1	
Inra-abdominal fluid collection or abscess (after surgery)		2	
Wound complication	2		
Abscess recur	2		
PCD insertion site abscess	1		
Mechanical ileus		2	

IA indicates interval appendectomy; PCD, percutaneous catheter drainage.

## DISCUSSION

Recently, there have been several well-designed studies on uncomplicated appendicitis treatment with antibiotics only. In addition, nonoperative management of early appendicitis with no complication showed an acceptable control rate compared with operative management.^8^–^11^ However, laparoscopic appendectomy is still considered the standard treatment for uncomplicated acute appendicitis.^12^

There are still controversies in performing surgery for patients with abscess from perforated appendicitis. Usually, the surgeon considers the patient’s conditions and the surgeon’s expertise and confidence when they are selecting the way of treatment. In case a patient has severe associated diseases or bowel adhesions, surgeons may decide to treat that case with PCD assisted by interventional imaging and antibiotics.^7^ Surgeons may consider conservative management to allow time for symptoms to resolve or to treat comorbidities in complex patients and afterward decide for IA. IA is recommended 8 to 12 weeks after conservative management.^13^ The delay in the surgery provides time for the inflammation to subside; hence, the procedure is considered safer and easier.^14^ Patients were also studied with colonoscopy or barium enema since inflammatory bowel disease or colon cancer can sometimes be hidden under perforated appendicitis.^15^

Direct surgery for perforated appendicitis with abscess is generally avoided because of a high rate of intra-abdominal abscess recurrence and operation site infections, and the difficulties in closing the appendiceal stump. The unclosed or re-opened stump could lead to a recurrence of abscesses.^16^ Thus, open surgery is preferred to control the abscess.^17^ The morbidity rate of patients with intraperitoneal abscesses increases as the infection rate of the surgical site and the length of patient hospitalization also increase. Additional medical support and imaging expertise for patients also increase costs.^17^

However, if the rate of complication between IA after medical care and direct operation with an abscess is not different, then DS could be recommended as the best intervention for complicated appendicitis. Some studies support direct surgery over nonoperative management for complicated appendicitis. Blakely et al^18^ observed higher rates of adverse events, such as intra-abdominal abscess, small bowel obstruction, wound infection, unplanned readmissions, central venous line-related complications, and recurrent appendicitis in patients who underwent IA.^18^ St. Peter et al^19^ found that there were no significant differences in the total length of hospitalization and recurrent abscess rate after treatment, between the groups.^19^ Furthermore, patients in the nonoperative group were subjected to more hospital visits and diagnostic tests. In contrast, the direct surgery group patients showed the advantage of a short length of hospitalization. Blakely et al^18^ also showed that nonoperative patients underwent 38% more CT scans than direct surgery patients. It could elevate patients’ radiation exposure risk equivalent to 25.7 months of natural background radiation exposure in one time of A-P CT.^18^

We analyzed the cost-effectiveness of the techniques in both groups. Our results showed that patients who underwent IA after medical treatment stay at the hospital and visit the OPD more than those who underwent direct surgery. Moreover, 72% of patients in the IA group underwent a CT scan twice to evaluate the results of medical care. The IA group patients had to pay ~1.6 times the hospital stay cost and 8 times the OPD visit cost compared with the direct surgery group. However, there was no significant difference between the 2 groups regarding postmanagement complications.

The main limitations of this study were the retrospective study design and the small sample size. Also, even this is a single-center study but there are over 10 surgeons who are on-duty. It means that the surgeons who treated the patients were different so the method of treatment could not be unified. Because the method of treatment mainly depends on the surgeon’s decision. A well-designed study could overcome these limitations.

## CONCLUSION

Direct surgery for perforated appendicitis accompanied by abscess is one of the choices for the management of complicated appendicitis patients. Morbidity and mortality risks associated with direct surgery is not significantly higher than those associated with IA after PCD management. Moreover, direct surgery is more cost-effective than IA.
